# Evaluating implementation of methicillin-resistant *Staphylococcus aureus* (MRSA) prevention guidelines in spinal cord injury centers using the PARIHS framework: a mixed methods study

**DOI:** 10.1186/s13012-015-0318-x

**Published:** 2015-09-09

**Authors:** Salva N. Balbale, Jennifer N. Hill, Marylou Guihan, Timothy P. Hogan, Kenzie A. Cameron, Barry Goldstein, Charlesnika T. Evans

**Affiliations:** Spinal Cord Injury Quality Enhancement Research Initiative, Edward Hines Jr. VA Hospital, US Department of Veterans Affairs, Hines, IL USA; Center of Innovation for Complex Chronic Healthcare, Edward Hines Jr. VA Hospital, US Department of Veterans Affairs, Hines, IL USA; Center for Healthcare Studies, Institute of Public Health and Medicine, Northwestern University Feinberg School of Medicine, Chicago, IL USA; Department of Physical Medicine and Rehabilitation, Northwestern University Feinberg School of Medicine, Chicago, IL USA; Center for Healthcare Organization and Implementation Research, Edith Nourse Rogers Memorial Veterans Hospital, US Department of Veterans Affairs, Bedford, MA USA; eHealth Quality Enhancement Research Initiative, National eHealth QUERI Coordinating Center, Edith Nourse Rogers Memorial Veterans Hospital, US Department of Veterans Affairs, Bedford, MA USA; Division of Health Informatics and Implementation Science, Department of Quantitative Health Sciences, University of Massachusetts Medical School, Worcester, MA USA; Division of General Internal Medicine and Geriatrics, Department of Medicine, Northwestern University Feinberg School of Medicine, Chicago, IL USA; Department of Preventive Medicine, Northwestern University Feinberg School of Medicine, Chicago, IL USA; Patient Care Services, Spinal Cord Injury/Disorders Services, US Department of Veterans Affairs, Seattle, WA USA; Department of Rehabilitation Medicine, University of Washington, Seattle, Washington USA

## Abstract

**Background:**

To prevent methicillin-resistant *Staphylococcus aureus* (MRSA) in Spinal Cord Injury and Disorder (SCI/D) Centers, the “Guidelines for Implementation of MRSA Prevention Initiative in the Spinal Cord Injury Centers” were released in July 2008 in the Veterans Affairs (VA) Health Care System. The purpose of this study was to use the Promoting Action on Research Implementation in Health Systems (PARiHS) framework to evaluate the experiences of implementation of SCI/D MRSA prevention guidelines in VA SCI/D Centers approximately 2–3 years after the guidelines were released.

**Methods:**

Mixed methods were used across two phases in this study. The first phase included an anonymous, web-based cross-sectional survey administered to providers at all 24 VA SCI/D Centers. The second phase included semi-structured telephone interviews with providers at 9 SCI/D Centers. The PARiHS framework was used as the foundation of both the survey questions and semi-structured interview guide.

**Results:**

The survey was completed by 295 SCI/D providers (43.8 % response rate) from 22 of the 24 SCI/D Centers (91.7 % participation rate). Respondents included nurses (57.3 %), therapists (24.4 %), physicians (11.1 %), physician assistants (3.4 %), and other health care professionals (3.8 %). Approximately 36 % of the SCI/D providers surveyed had not seen, did not remember seeing, or had never heard of the MRSA SCI/D guidelines, whereas 42.3 % of providers reported that the MRSA SCI/D guidelines were fully implemented in their SCI/D Center. Data revealed numerous barriers and facilitators to guideline implementation. Facilitators included enhanced leadership support and provider education, focused guideline dissemination to reach SCI/D providers, and strong perceived evidence supporting the guidelines. Barriers included lack of awareness of the guidelines among physical therapists and physician assistants and challenges in cohorting/isolating MRSA-positive patients and following contact precautions.

**Conclusions:**

Successful implementation of MRSA infection prevention guidelines in SCI/D settings requires (1) guideline dissemination that reaches the full range of SCI/D providers working in inpatient, outpatient, and other care settings, (2) provider education that is frequent and systematic, (3) strong leadership support, and (4) that barriers unique to the recommendations are addressed. These findings may be used to inform selection of implementation strategies and optimize infection prevention beyond MRSA as well as in other specialty care populations.

**Electronic supplementary material:**

The online version of this article (doi:10.1186/s13012-015-0318-x) contains supplementary material, which is available to authorized users.

## Background

Methicillin-resistant *Staphylococcus aureus* (MRSA) is responsible for over 11,000 deaths and 200,000 hospitalizations in the USA every year, in addition to increases in health care costs and length of stay [1–4]. In 2007, the Veterans Affairs (VA) Health Care System released the MRSA Prevention Initiative [5], describing infection control strategies to prevent the spread of MRSA in VA medical centers. This initiative used a bundled intervention strategy and resulted in decreased MRSA transmission and infection in the VA [6]. Components of the initiative included (1) active surveillance and screening cultures (ASCs), (2) use of contact precautions by health care providers if patients are found to be MRSA positive, (3) hand hygiene, and (4) cultural transformation through staff and leadership engagement [5]. The success of this initiative has been presented elsewhere including declines in MRSA infection rates in intensive care unit (ICU) and non-ICU settings [6, 7].

Additional guidance was provided for prevention of MRSA in VA Spinal Cord Injury and Disorder (SCI/D) Centers. Patients with chronic and complex health needs, such as those with SCI/D, are at particularly high risk for MRSA and other hospital-acquired infections (HAI) [7–9]. The “Guidelines for Implementation of MRSA Prevention Initiative in the Spinal Cord Injury Centers” (herein referred to as SCI/D MRSA prevention guidelines) were released in July 2008 [10]. These guidelines (Additional file 1) addressed specific elements of SCI/D care, such as the inclusion of wounds and/or pressure ulcers as part of ASCs. The SCI/D MRSA prevention guidelines also emphasized hand hygiene in patients and providers before and after urinary catheterization and bowel care, addressed appropriate cleaning of shared equipment, and highlighted the need for contact precautions and environmental cleaning. The initiative was based on evidence from the Centers for Disease Control around management of multidrug-resistant organisms in health care settings [11]. Although research evidence clearly supports the components of the guidelines related to hand hygiene, contact precautions, and the presence of leadership and culture change [12, 13], the strength of evidence underlying the universal screening and isolation strategy is less clear [14]. Similar to the ICU and non-ICU settings, significant decreases in MRSA infection rates have been seen in VA SCI/D Centers [7].

Given the success of the MRSA Prevention Initiative in VA overall and in SCI/D Centers [6, 7], identifying barriers and facilitators that impacted early guideline implementation in SCI/D Centers is a critical step to enhance implementation of future prevention initiatives that are rolled out. Successful infection prevention depends on effective implementation of evidence-based guidelines [15, 16]. However, translating infection prevention guidelines into practice in SCI/D Centers can be especially challenging due to several characteristics unique to patients with SCI/D and their needs and experiences [7, 9]. Specifically, patients with SCI/D are more susceptible to MRSA colonization and infection due to frequent and prolonged hospitalization [9, 17], frequent contact with body fluids (e.g., during bowel programs and bladder catheterization), high prevalence of pressure ulcers [18, 19], and extensive antibiotic use due to infections such as urinary tract infections and pneumonia [9, 20]. Controlling the spread of MRSA in hospital settings where individuals with SCI/D are present is further complicated by the use of common areas for rehabilitation and exercise, shared equipment, and difficulties in performing hand hygiene among patients with impaired hand function secondary to tetraplegia.

Previous studies on guideline implementation have demonstrated that without systematic and tailored implementation strategies, little impact is observed on routine clinical practice [21–23]. Prior implementation studies also have demonstrated the importance of using theory-based approaches or conceptual frameworks, such as the Promoting Action on Research Implementation in Health Systems (PARiHS), to comprehensively evaluate implementation [16, 24–26]. The purpose of this study was to use the PARiHS framework to evaluate implementation of the SCI/D MRSA prevention guidelines in VA SCI/D Centers approximately 2–3 years after the guidelines were released.

## Methods

### Study design and participants

Quantitative and qualitative approaches were used across two phases in this mixed methods study. We focused on providers’ experiences with early implementation of the SCI/D MRSA prevention guidelines and recommended practices, approximately 2–3 years following the release of the guidelines. Additionally, we assessed provider perceptions and familiarity with the guidelines, factors influencing early implementation, and attitudes on prevention practices and perceived strength of evidence around MRSA severity and prevention.

This study was focused on the 24 regional SCI/D Centers that were part of the VA SCI/D System of Care at the time of the study. The first phase of the study included an anonymous, web-based cross-sectional survey administered in August 2010 to providers at all 24 VA SCI/D Centers. The sampling frame was identified through VA electronic mailing lists (LISTSERVs) of providers working in SCI/D Centers and a contact list of MRSA Prevention Coordinators (MPCs) across the country. Local MPCs maintain responsibility for ensuring implementation of the MRSA Initiative at each VA inpatient facility. Survey data were collected as part of a broader effort to characterize MRSA prevention practices in SCI/D Centers following guideline release. Although the initial sample of providers was composed of MPCs at each Center’s facility, as well as the SCI/D Center Chief, SCI/D Center physicians, nurses, therapists, and other allied health providers, we only describe survey data from the SCI/D Center physicians, nurses, therapists, and allied health providers. MPC survey data were omitted from our analysis as these data did not focus on perceptions of guideline implementation.

The second phase of the study included semi-structured telephone interviews with providers at nine of the SCI/D Centers, which occurred December 2010–March 2011. The research team selected interview questions about current practices that were critical to guideline implementation [27]. Participants were recruited through the SCI/D Chief, Infection Control Coordinator, MPC, or other designated leaders at each of the nine VA facilities. Background on the objectives of the study was provided, and permission to interview providers requested. A recruitment letter about the study also was distributed to potential participants.

This study was approved by the Institutional Review Board at the Hines VA Hospital. Further details on the study design and data collection efforts are published elsewhere [27].

### Conceptual framework

The PARiHS framework was used as the foundation of both the survey questions and semi-structured interview guide (Additional file 2: Appendix A). The PARiHS framework proposes that successful implementation of evidence into practice is a function of three broad interactive elements: evidence, context, and facilitation [28–30]. Given the lack of knowledge around infection prevention guideline implementation in SCI/D, we focused on building our understanding around contextual factors that affected early implementation of the SCI/D MRSA prevention guidelines at each VA SCI/D Center, with a secondary focus on evidence and facilitation factors influencing early implementation.

### Survey procedures and instrument

Catapult Systems Corporation’s InquisiteWeb Survey System 7.0 was used to administer the anonymous survey. Respondents were provided with an electronic link through an email invitation generated by the system; the survey was available for 3 weeks. Two reminder emails were sent during this period to those who had not completed the survey.

The survey included questions designed to explore early implementation of the SCI/D MRSA prevention guidelines as well as demographic questions, including type of provider, age, number of years in training, VA facility, and supervisory status. Questions explored attributes known to influence guideline adoption [29–31], including (1) providers’ perceived strength and extent of evidence underlying the guidelines, (2) the quality of the organizational context for guideline implementation, and (3) facilitation efforts to implement the SCI/D MRSA prevention guidelines.

### Semi-structured interview procedures

Qualitative data were collected to examine the factors shaping provider survey responses and guideline implementation. Survey data were used to select the study sites for interview [27]. The research team selected survey questions to gather information about frequency of provider engagement in practices from the guidelines that are critical for MRSA prevention. These practices include conducting active surveillance, placing MRSA-positive patients on contact precautions, and educating patients and caregivers on MRSA prevention. The percentage of survey respondents from each SCI/D Center who reported “usually/always” performing these prevention practices was used to create a summary score for their respective SCI/D Center. We used these summary scores to organize facilities into low, medium, and high scores. A sample of 2–4 SCI/D Centers was selected from each of these groups for a total of nine study sites. To ensure adequate representation from leadership within the SCI/D Center and infection control, interviewees included at least one designated leader in the SCI/D Center for the MRSA program and the MPC or another infection control representative. We recruited up to four participants at each facility. Interview participants were unaware of the selection process.

Four authors (JNH, TPH, KAC, and MG) trained in qualitative data collection conducted the interviews. Informants were first asked to respond to a short set of background questions regarding demographics, professional training, and work experience. A one-on-one, semi-structured, audio-recorded interview followed, lasting from 30 to 60 min. Interview questions were related to perceptions of early efforts to implement the SCI/D MRSA prevention guidelines and the range of facilitating or impeding factors influencing their implementation.

### Analysis

All survey questions were described using univariate statistics (frequencies, percentages, and means) and chi-square statistics. Analysis of perceived level of implementation and guideline awareness across provider types involved multiple comparisons; to minimize the possibility of type I error, we applied a Bonferroni-corrected alpha level as the criteria of statistical significance for these tests. We used an alpha level of 0.017 for comparisons of perceived level of implementation across provider groups and an alpha level of 0.008 to compare guidelines awareness across provider groups. All analyses were conducted using SAS software version 9.2 (SAS Institute Inc, Cary, NC, USA).

Audio-recorded interviews were transcribed verbatim and compared with the original recordings to ensure transcription accuracy. All personal identifiers were removed, and the transcripts were distributed to the coders. Transcripts were analyzed using a constant comparative technique to organize the content into categories, identifying both common and unique themes across interviews [32, 33]. A preliminary coding scheme was developed, and interviews were independently coded by four research study members (JNH, TPH, KAC, and MG), where each interview was independently coded by at least two study members. Discrepancies in coding were further refined based on discussions between the coders. In line with the PARiHS model, we explored the different elements of context, evidence, and facilitation associated with guideline implementation across the nine sites as well as provider perceptions related to implementation of the SCI/D MRSA prevention guidelines.

## Results

### Quantitative survey

The survey was completed by 295 SCI/D providers (43.8 % response rate) from 22 of the 24 SCI/D Centers (91.7 % participation rate). Respondents were excluded in cases of (1) incomplete surveys, (2) undeliverable responses due to termination of VA employment, and (3) providers who did not treat patients with SCI/D or were not involved in direct patient care. Thus, our final eligible sample size included 234 potential respondents (34.8 % response rate). Respondents included nurses (57.3 %), therapists (24.4 %), physicians (11.1 %), physician assistants (3.4 %), and other health care professionals (3.8 %). The percentage of each provider type who responded was 40.1 % of nurses, 16.8 % of physicians, 42.1 % of physician assistants, 33.5 % of therapists, and 4.7 % of other health care professionals. Respondents cared for SCI/D patients in inpatient, outpatient, long-term care, and home care settings. Table 1 includes demographic characteristics of the responding SCI/D Center providers. Although responses to the survey were anonymous, we compared the original sampling frame to those that responded by provider type [27]. When comparing survey respondents to the original sampling framework, the distribution of respondents was similar for nurses, therapists, and physician assistants. However, physicians were less likely to participate in the survey (11 % of respondents compared to 23 % of the sampling frame).

**Table 1:** Provider demographic characteristics

SCI/D providers		*n* = 234	
		Frequency (%)	
Type of SCI/D unit setting worked in most of the time			
Long-term care		23 (9.8)	
Inpatient unit		177 (75.7)	
Outpatient clinic		27 (11.5)	
Home care		7 (3.0)	
Gender			
Male		56 (24)	
Female		178 (76)	
Provider type			
Nurse		134 (57.3)	
Therapist		57 (24.4)	
Physician		26 (11.1)	
Other		9 (3.8)	
Physician assistant		8 (3.4)	
Type of nurse^a^		*n* = 81	
Bachelor of Science Registered Nurse (BSRN)		36 (44.4)	
Licensed Practical Nurse (LPN)		21 (25.9)	
Master of Science—Nursing (MSN)		7 (8.7)	
Nurse Practitioner (NP)		6 (7.4)	
Other^b^		11 (13.6)	
Type of therapist		*n* = 57	
Occupational therapist		21 (36.8)	
Physical therapist		18 (31.6)	
Therapeutic recreation therapist		11 (19.3)	
Other^c^		7 (12.3)	
Percentage of time spent on direct patient care^a^		*n* = 226	
Average time spent		64.7 %	
Range: 0–100 %			
Average number of years working with patients with SCI/D*		10.9 years	
Range: 1–40 years			
SCI/D provider characteristics by perceived degree of implementation	*n* = 228		
	Frequency (%)		
	Fully implemented	Not fully implemented	*p* value (chi-square)
Position at the VA			0.035
Nurse	63 (65.63)	66 (50)	Ref
Physician	11 (11.46)	15 (11.36)	0.544
All others	22 (22.92)	51 (38.64)	0.010
Type of SCI/D unit setting worked in primarily			0.506
Home care	1 (1.04)	4 (3.03)	
Inpatient unit	78 (81.25)	97 (73.48)	
Long-term care	8 (8.33)	14 (10.61)	
Outpatient clinic	9 (9.38)	17 (12.88)	
Gender			0.584
Male	75 (78.13)	99 (75)	
Female	21 (21.88)	33 (25)	

^a^Indicates missing values

^b^Includes clinical educators and certified nursing assistant (CNA)

^c^Includes kinesiotherapists and respiratory therapists

Overall, 42.3 % of providers reported that the SCI/D MRSA prevention guidelines were fully implemented in their SCI/D Center, followed by 47.0 % that indicated mostly, and 10.7 % that they were partially implemented. Across the 22 sites, perceptions of full implementation of the guidelines ranged from 0–100 %. Nurses were significantly more likely to report full implementation of the guidelines compared to other positions at the Bonferroni-corrected alpha level of *p* < 0.017 but were similar in comparison with physicians (Table 1); no significant difference was found between physicians and other positions (*p* = 0.258). Perceived level of implementation did not differ significantly across levels of nurse education.

Table 2 reports respondents’ familiarity with the SCI/D MRSA prevention guidelines, perceptions around implementation of the guidelines, and specific prevention practices in their SCI/D Center, by perception of full implementation. Compared to those who did not, staff members who perceived the guidelines to be fully implemented were more likely to endorse other factors that contribute to guideline implementation such as being aware of the guidelines, having resources to implement the guidelines including provider support, training and funding, and perceiving a beneficial impact on prevention of MRSA transmission and infection in patients with SCI/D. Those who reported full implementation were also more likely to report hygiene practices such as bathing patients with SCI/D every day and encouraging hand hygiene in visitors/family members and patients. Barriers to cohorting patients or patient hand hygiene were not associated with perception of implementation; however, some differences in barriers were noted for providers in changing gowns and gloves in-between patients. Multi-bed rooms, expense of gowns and glove supplies, and providers not following guidelines were more frequently reported in those who felt that the guidelines were not fully implemented.

**Table 2:** Provider survey responses, by perceived degree of implementation

	*n* = 228		
	Frequency (%)		
	Fully implemented	Not fully implemented	*p* value (chi-square)
Have seen the VA guidelines for MRSA prevention in SCI/D Centers	76 (79.17)	69 (52.27)	<0.0001
Agree^a^ that your SCI/D center/interdisciplinary team works closely with the hospital MRSA Prevention Coordinator (MPC) to prevent MRSA transmission	72 (75)	85 (64.39)	0.0877
There is a person or a group of people (aside from the hospital MPC) responsible for implementation of SCI/D Center MRSA Program guidelines in your unit	69 (71.88)	75 (56.82)	0.02
Agree^a^ that *staff* resources provided by the SCI/D Chief to implement the SCI/D MRSA Program are adequate	79 (84.04)	75 (61.98)	0.0004
Agree^a^ that *training* resources provided by the SCI/D Chief to implement the SCI/D MRSA Program are adequate	76 (81.72)	76 (63.87)	0.0042
Agree^a^ that *funding* resources provided by the SCI/D Chief to implement the SCI/D MRSA Program are adequate	45 (70.31)	41 (43.16)	0.0008
Agree^a^ that implementation of the SCI/D Center MRSA Program guidelines has greatly improved your ability to prevent MRSA transmission to MRSA-negative patients in the SCI/D unit	71 (73.96)	65 (49.24)	0.0002
Agree^a^ that implementation of the SCI/D Center MRSA Program guidelines has greatly improved your ability to prevent MRSA infection in *all* SCI/D patients	65 (67.71)	58 (43.94)	0.0004
Usually or always^b^ indicate in a signed/dated progress note that communication with a patient about MRSA screening has occurred	56 (65.12)	39 (34.82)	<0.0001
Frequency of practicing the following in your SCI/D center			
Usually or always^b^ encourage visitors/family members to perform hand hygiene before/after all patient contact	82 (88.17)	86 (68.25)	0.0006
Usually or always^b^ encourage SCI/D patients to perform hand hygiene before/after interacting with other patients	80 (84.21)	79 (61.72)	0.0002
Usually or always^b^ encourage SCI/D patients with impaired hand function to ask for assistance to perform hand hygiene	82 (86.32)	82 (64.57)	0.0003
Usually or always^b^ bathe SCI/D patients every day	31 (43.66)	29 (28.16)	0.0344
Agree^a^ that health care worker hand hygiene is an effective way to reduce MRSA transmission and the development of MRSA infections in SCI/D patients	94 (97.92)	125 (94.7)	0.2177
Agree^a^ that I feel comfortable reminding other staff in the SCI/D unit when they do not clean their hands	74 (77.08)	79 (59.85)	0.0062
Agree^a^ that patient hand hygiene is an effective way to reduce MRSA transmission and development of MRSA infections in SCI/D patients	91 (94.79)	113 (85.61)	0.0257
Agree^a^ that MRSA colonization can be prevented in SCI/D patients	66 (70.97)	74 (62.71)	0.2076
Agree^a^ that MRSA transmission in hospitalized SCI/D patients can be prevented	77 (81.05)	92 (73.6)	0.1945
Barriers to cohorting/isolating MRSA-positive patients			
Availability of beds	63 (65.63)	85 (64.39)	0.8475
Too many MRSA-positive patients	60 (62.5)	72 (54.55)	0.2297
Layout of unit	24 (25)	47 (35.61)	0.0877
Patients refuse or get upset	20 (20.83)	34 (25.76)	0.3879
Visitors/family members refuse or get upset	21 (21.88)	37 (28.03)	0.292
Too busy	7 (7.29)	9 (6.82)	0.8901
Delayed time to get results	13 (13.54)	18 (13.64)	0.9836
Disrupts work flow of unit	15 (15.63)	26 (19.7)	0.4293
Other	10 (10.42)	24 (18.18)	0.1041
Barriers to patient hand hygiene in your SCI/D unit			
Not enough staff available to assist	19 (19.79)	34 (25.76)	0.2924
Inadequate availability of wheelchair accessible sink with soap and water	9 (9.38)	22 (16.67)	0.1127
Inadequate availability of touchless soap or hand sanitizer	16 (16.67)	34 (25.76)	0.1014
Patient unwilling to perform hand hygiene	52 (54.17)	85 (64.39)	0.1195
Other	21 (21.88)	27 (20.45)	0.7951
Barriers to changing gown and gloves in-between patients			
SCI/D unit layout	9 (9.38)	31 (23.48)	0.0057
Competing demands/too busy	27 (28.13)	38 (28.79)	0.9128
Multi-bed rooms	37 (38.54)	71 (53.79)	0.0228
Expense of supplies	3 (3.13)	17 (12.88)	0.0102
Staff do not follow the SCI/D Center MRSA Program guidelines	15 (15.63)	37 (28.03)	0.0275
Other	20 (20.83)	33 (25)	0.4621

^a^Agree = Strongly agree/agree versus neutral/disagree/strongly disagree

^b^Usually/always = usually/always versus sometimes/rarely/never

Perceived strength of research evidence among providers was also assessed (Table 3). Most providers perceived evidence underlying the SCI/D MRSA prevention guidelines to be strong. Perceived strength of evidence was generally stronger in the fully implemented group for several key MRSA prevention practices, including active surveillance measures at admission, hand hygiene, and gowning and glowing before entering a MRSA patient’s room.

**Table 3:** Perceived strength of evidence among providers, by perceived degree of implementation

	*n* = 228		
	Frequency (%)		
	Fully implemented	Not fully implemented	*p* value (chi-square)
Perceived strength of evidence for prevention of MRSA transmission in SCI/D patients for the following practices			
Gloving and gowning before entry into a MRSA-positive patient’s room			<0.0001
Very strong/strong	86 (89.58)	86 (65.15)	
Neutral/weak/very weak/don’t know	10 (10.42)	46 (34.85)	
Taking off gloves and gown before leaving a patient’s room.			0.0001
Very strong/strong	88 (91.67)	94 (71.21)	
Neutral/weak/very weak/don’t know	8 (8.33)	38 (28.79)	
That anyone entering a MRSA-positive patient’s room should wear gown and gloves			<0.0001
Very strong/strong	80 (83.33)	68 (51.52)	
Neutral/weak/very weak/don’t know	16 (16.67)	64 (48.48)	
Perceived strength of evidence for handwashing to prevent the spread of resistant organisms (including MRSA) in SCI/D			
Health care workers before and after patient contact			0.012
Very strong/strong	90 (93.75)	109 (82.58)	
Neutral/weak/very weak/don’t know	6 (6.25)	23 (17.42)	
Visitors/family members before and after patient contact			0.001
Very strong/strong	79 (82.29)	83 (62.88)	
Neutral/weak/very weak/don’t know	17 (17.71)	49 (37.12)	
SCI/D patients			0.004
Very strong/strong	73 (76.04)	76 (57.58)	
Neutral/weak/very weak/don’t know	23 (23.96)	56 (42.42)	
SCI/D patients with poor hand function			0.044
Very strong/strong	63 (65.63)	69 (52.27)	
Neutral/weak/very weak/don’t know	33 (34.38)	63 (47.73)	
Perceived strength of evidence that supports the following practices in SCI/D units			
Active surveillance at admission			0.002
Very strong/strong	79 (82.29)	84 (63.64)	
Neutral/weak/very weak/don’t know	17 (17.71)	48 (36.36)	
Active surveillance at transfer to another unit			<0.0001
Very strong/strong	76 (79.17)	69 (52.27)	
Neutral/weak/very weak/don’t know	20 (20.83)	63 (47.73)	
Active surveillance at discharge			0.0001
Very strong/strong	76 (79.17)	72 (54.55)	
Neutral/weak/very weak/don’t know	20 (20.83)	60 (45.45)	
Active surveillance of wounds or pressure ulcers			0.0004
Very strong/strong	73 (76.04)	70 (53.03)	
Neutral/weak/very weak/don’t know	23 (23.96)	62 (46.97)	
Rescreening during long admissions			<0.0001
Very strong/strong	67 (69.79)	57 (43.18)	
Neutral/weak/very weak/don’t know	29 (30.21)	75 (56.82)	

Table 4 describes awareness of the SCI/D MRSA prevention guidelines by provider characteristics. Of the SCI/D providers surveyed, 36.8 % had not seen, did not remember seeing, or had never heard of the SCI/D MRSA prevention guidelines. A smaller proportion of physical therapists and assistants reported being aware of the guidelines compared to nurses. We did not find a statistically significant difference in guideline awareness across provider types. Guideline awareness was strongest among providers in the initial years of their experience in working with SCI/D patients. There was no difference in awareness across years of experience when stratified by provider type.

**Table 4:** Awareness of SCI/D MRSA prevention guidelines, by provider characteristics

	*n* = 228		
	Frequency (%)		
	Awareness of SCI/D MRSA prevention guidelines		
Provider characteristic	Aware of guidelines	Not aware of guidelines	*p* value (chi-square)
All providers	144 (63.2)	84 (36.8)	
Gender			0.664
Male	33 (66.1)	21 (38.9)	
Female	112 (64.4)	62 (35.6)	
Provider type			0.238
Nurse	89 (69.0)	40 (31.0)	Ref
Physician	16 (61.5)	10 (38.5)	0.458
Physician assistant/therapist	35 (55.6)	28 (44.4)	0.068
Other	5 (50)	5 (50)	0.216
Experience^a^ (*n* = 186)			0.026
0–2 years	23 (85.2)	4 (14.8)	
3–5 years	23 (63.9)	13 (36.1)	
6–8 years	14 (43.8)	18 (56.3)	
9 years or more	57 (62.6)	34 (37.4)	
Type of SCI unit			0.009
Inpatient unit	121 (69.1)	54 (30.9)	Ref
Outpatient clinic	14 (53.9)	12 (46.1)	0.121
Long-term care	8 (36.4)	14 (63.6)	0.002
Home care	2 (40.0)	3 (60.0)	0.167

^a^Indicates missing values

### Qualitative themes

Thirty interviews were conducted across nine SCI/D Centers in the second phase of the study. The final make-up of the interview sample (*n* = 30) included 16 nurses (8 registered nurses, 7 Bachelor of Science registered nurses, 1 certified nursing assistant), 9 physicians, 4 physical therapists, and 1 physician assistant. Three of the nurses were also MPCs. We used the qualitative interview data to examine the provider perspective and better understand providers’ survey responses. Prominent themes emerging from the data included provider awareness of the SCI/D MRSA prevention guidelines, perceived evidence supporting the guidelines, as well as leadership support and resources to facilitate implementation of the guidelines. Themes were grouped according to the PARiHS constructs (context, evidence, and facilitation) to explore factors influencing guideline implementation in VA SCI/D Centers (Additional file 3: Appendix B). Key survey results were linked with our qualitative findings to triangulate our data and interpret their relevance to implementation.

### Context

Awareness of the SCI/D MRSA prevention guidelines varied significantly across the nine sites, where some providers had greater familiarity and others reported limited or no exposure to them. Based on survey findings, approximately 64 % of SCI/D providers surveyed were aware of the guidelines. Guideline awareness was generally higher among providers who perceived the guidelines to be fully implemented, compared to those who felt the guidelines were not fully implemented. Interview data revealed possible factors within the receptive context that may be driving a lack of guideline awareness, including gaps in the dissemination of the guidelines.

Providers reported varied experiences in learning about and accessing the SCI/D MRSA prevention guidelines. Guideline dissemination did not always reach all providers systematically. Among SCI/D providers interviewed, physical therapists and physician assistants generally reported not learning about the guidelines. One physical therapist who had not read the guidelines reported: “Everything that I’ve seen is second hand information that I am told through education…but as far as reading the [SCI/D MRSA prevention guidelines] policy myself, no [I have not].” Another physical therapist noted a disconnection in awareness of the guidelines between newer providers and those who had worked in a VA SCI/D Center longer.

Other providers were very familiar with the guidelines and were able to access them frequently. One SCI/D nurse said: “I’m pretty familiar [with the SCI/D MRSA prevention guidelines]…and I actually have it on my desktop always.” Others reported that they had some familiarity with the guidelines but did not have in-depth knowledge around them and had not reviewed them recently. An SCI/D nurse reported: “I’m not like the clinicians where I know it by heart or anything like that. I know the basic process.”

Another key component of the receptive context of implementation was a sense of receptivity to change among providers. This often included practices and strategies initiated by providers to better implement practices from the SCI/D MRSA prevention guidelines. One physician noted one example of such an activity to promote patient hand hygiene: “[Providers] getting hand sanitizer for patients at the bedside because they know bed-bound patients do self-care.”

Providers commented on contextual aspects specific to the culture within their SCI/D Centers and MRSA prevention program. One nurse/MPC described a prevailing belief within her SCI/D Center to be more lenient with respect to implementation of the SCI/D MRSA prevention guidelines, compared to acute care settings where she perceived a greater need for MRSA prevention: “It’s… acute care where we want [MRSA prevention] to be the most stringent; and then spinal cord injury is a little more lenient.” In addition, one nurse commented on steps her SCI/D Center had taken to promote learning and adoption of contact precautions, as well as to improve patient and provider safety: “We spearheaded… and developed isolation signage that is consistent…colors, verbiage and everything. That’s how we present isolation. When we teach it, it’s like ‘this is for your protection and also the protection of the multiple patients you care for.’”

Evaluation, another contextual sub-element, was also discussed. Specifically, providers reflected on individual- and system-level feedback received on their performance if they were effectively implementing prevention practices and also if there was a need to improve implementation of specific prevention practices. One nurse commented: “I do get feedback from [SCI/D leadership]. When [staff] do really great, we get very good feedback…. If they’re not doing so great, we get support from leadership saying ‘this is important,’ ‘please do the swabs,’ ‘make sure you adhere to contact precautions,’ or ‘continue educating patients on the importance of hand hygiene.’”

### Evidence

In terms of research evidence, providers generally perceived strong evidence for the prevention practices listed in the SCI/D MRSA prevention guidelines, including hand hygiene, contact precautions, and active surveillance. Findings from the survey showed that providers who perceived the guidelines to be fully implemented also perceived stronger evidence supporting the guidelines, as compared with providers who did not perceive full implementation.

Interview data highlighted practices that providers perceived to be particularly important for MRSA prevention in SCI/D, often corresponding with survey responses. Hand hygiene was also associated with strong or very strong perceived research evidence in the survey data. One physician stated: “The most important, in my opinion, is that [providers] follow the hand washing…that’s the number one thing we can do to prevent the spread of [MRSA].” Other areas with strong perceived evidence included contact precautions and active surveillance in SCI/D Centers. One nurse stated: “[Providers] need to know the precautions, to make sure [patients] are put on contact precautions immediately to prevent the spread of MRSA.” A nurse stated: “Certainly [active] surveillance is important…because it gives us the power to get patients on contact isolation in a timely matter.” One nurse/MPC commented: “Hand hygiene and isolation is always important….[And] patients participating in education and performing hand hygiene. I think that is important in spinal cord [injury].”

Clinical experiences were often viewed by providers as evidence supporting the SCI/D MRSA prevention guidelines. Providers reflected on their own clinical experiences around MRSA prevention, many noting that they found specific guidelines for contact precautions and active surveillance to be especially relevant for MRSA prevention. Based on their experiences, many providers also believed that MRSA was the most problematic infection-causing organism in their SCI/D Center. One physician stated: “It’s just by my clinical experience [in SCI/D] that I have had more issues with patient having MRSA…it’s just relative to the other bugs that I seem to have experience with.”

Interview data also revealed possible contextual factors in knowledge that may explain weaker perceived evidence in the “not fully implemented” group. When asked to comment on the importance of components of the guidelines, one nurse stated: “It’s hard for me to comment on that because I’m just not really sure.” A nurse/MPC commented: “I used to be pretty familiar [with the guidelines] at least when they were first released…I guess I probably can’t talk [about] the specifics.” In addition, some providers reported a lack of information from their own local context. One physician discussed the lack the data on “success rates” of one particular practice (swabbing of SCI/D patient wounds) included in the active surveillance component of the SCI/D MRSA prevention guidelines: “I don’t have data on the success rate of…[swabbing wounds], but the goal is that…all wounds are screened on admission.”

### Facilitation

Providers reported that efforts to facilitate implementation of the SCI/D MRSA prevention guidelines varied across the sites. These included leadership support, efforts to provide routine training and education, and to engage providers in the prevention initiative. Survey findings indicated that providers who perceived the guidelines to be fully implemented also believed that provider support, training, and funding resources were generally adequate to help implement the guidelines. Providers who felt the guidelines were not fully implemented often found these resources to be insufficient and, during the interviews, identified areas of improvement that could better facilitate implementation.

A key sub-element of facilitation was the role played by leadership to enable providers and support them in implementing the SCI/D MRSA prevention guidelines. Providers discussed several aspects of the facilitation role played by leadership at their SCI/D Center. One nurse stated: “[Infection Control and the SCI/D Chief] gave us a timeline on when these things had to be implemented, what we would need for each unit…how education was going to roll out to the staff…they gave us packets to be circulated. They were available online but you also had them if you wanted to put them in your hands. And they were going to do several education sessions about MRSA to staff. So they started setting that up and we would make sure all our staff attended to get an understanding of what we were doing.”

Other providers discussed leadership efforts that promoted adherence to the guidelines, such as placing hand hygiene stations and personal protective equipment in areas that would be most convenient for providers. One physical therapist commented: “[Leadership] makes sure that we have lots of contact precaution stuff out by every room.…That’s a huge help, making sure that they are stocked all the time so that people aren’t running around trying to find things….And they encourage people to comply.”

In addition, the role of internal agents within the SCI/D Center, such as MPCs and SCI/D leadership, as well as external agents, including infection control teams and broader hospital leadership, was a facilitator to support guideline implementation. One physical therapist commented on one of the roles played by infection control: “The Infection Control team comes in to make sure we have all the hand sanitizers readily available throughout the entire ward, throughout every clinic and room…They provide a lot more of the contact precaution carts and signs to alert all the staff that the patient is on contact [precautions] and have the equipment readily available to kind of increase compliance.” Efforts to monitor implementation of and compliance with the guidelines were also a facilitating role. Among the “fully implemented” group, a majority of providers reported that there was a person or group (aside from a hospital MPC) designated by leadership at their SCI/D Center to be responsible for implementing the guidelines, often improving compliance. One physician stated: “We have anonymous monitors on our ward. They monitor hand washing…they also monitor gowning and gloving and that’s reported as well. The leadership here…discuss…various ideas about how we can manage…maybe like drawing in an imaginary circle or maybe even a literal circle with tape on the floor around the bed and if you were to cross that you should gown.”

Finally, formal training organized and led by SCI Chiefs and MPCs was also an important facilitator to build providers’ initial awareness of the guidelines. These trainings used a didactic, traditional teaching approach delivered primarily through in-service sessions and educational materials tailored for SCI/D providers. While some providers learned about the SCI/D MRSA prevention guidelines directly from their MPCs/infection control or SCI/D Chiefs, others learned about them through VA education and training events facilitated by leadership. One nurse stated: “I [learned about the guidelines] from the MRSA Prevention Coordinator since the initiative started here…I got the initial directive when it came out.” Another nurse reflected: “Our SCI/D Chief had a meeting…and mentioned the guidelines. And then we may go and actually look at them ourselves.”

When providers indicated that leadership was not as actively involved, improvements suggested included a need for greater leadership involvement to increase provider buy-in and receptivity to the guidelines. One nurse/MPC indicated that “What we would need to make the [SCI/D MRSA] program more successful is more leadership support. There need to be consequences for not following the MRSA bundles.”

## Discussion

Few studies have explored the implementation of infection prevention guidelines in SCI/D. In this study, we combined the strengths of quantitative and qualitative methods [34–36] and used the PARiHS framework to evaluate efforts to implement the SCI/D MRSA prevention guidelines approximately 2–3 years following their release in VA SCI/D Centers. We focused on SCI/D provider perceptions, experiences, and insights into factors influencing implementation of the SCI/D MRSA prevention guidelines. Through this approach, we better understood the context in which SCI/D providers implement the guidelines and identified several implementation barriers and facilitators. If generalizable, lessons learned from this study may be used to optimize the continued implementation of MRSA infection prevention in SCI/D Centers, can be applied to enhance implementation strategies for other HAI prevention efforts in SCI/D, such as *C.difficile* infection prevention, and, finally, can be used as a model for evaluating implementation of guidelines in long-term care or other specialty care settings, such as polytrauma.

Variation in the implementation of the SCI/D MRSA prevention guidelines was reported across VA SCI/D Centers in this study, perhaps due to varying contextual factors at each site. Based on survey data, nearly 60 % of SCI/D providers believed that the SCI/D MRSA prevention guidelines were not fully implemented in VA SCI/D Centers. Several contextual and facilitation-related factors were explored in the interviews with providers to understand barriers and facilitators to implementation. These included variations in provider training, familiarity and awareness of guidelines, as well as organizational culture at SCI/D Centers, provider clinical experiences, and local leadership support and roles to facilitate implementation. Perceived evidence underlying the guidelines, although strong overall, was weaker among providers who did not perceive the guidelines to be fully implemented. Accounting for local contextual differences at SCI/D Centers will be important in designing future strategies to improve implementation of infection prevention guidelines in the SCI/D setting.

Challenges in implementing evidence-based guidelines are a well-documented problem in health care settings [22]. Prior studies have also highlighted the variability that exists in the implementation and subsequent success of HAI prevention programs [16, 37]. In SCI/D care delivery, a recent review has shown that knowledge translation and targeted implementation efforts may be particularly effective in changing SCI/D provider behavior and improving patient outcomes [38]. Our findings align with these studies and corroborate the need to increase provider awareness and knowledge around guidelines, ensure guidelines are tailored to the provider context, and enhance leadership support and provider education [16, 22, 39].

Provider perceptions and attitudes affect implementation of precautions to prevent transmission of drug-resistant pathogens such as MRSA, but a key lesson from this and other studies is that improving perceptions and attitudes alone may not be sufficient to fully implement best practices or guidelines for prevention [22, 40]. As evidenced in our work, barriers to implementation included lack of awareness of the guidelines and challenges in following contact precautions and cohorting and isolating MRSA-positive patients. Similarly, Seibert and colleagues found that adhering to hand hygiene and contact precautions was challenging for providers even though they felt that they had knowledge and ability to do so [40]. These barriers result in a lack of adherence to the guidelines in spite of provider knowledge, perceptions and motivation to follow them, and point to a disconnect between provider perceptions and prevention practices. This disconnect may explain some of the dissonant results we noted across perceived levels of implementation.

We identified perceived strengths and gaps in implementation that can be applied to future and ongoing initiatives. First, successful implementation of the SCI/D MRSA prevention guidelines requires strong leadership support and strong perceived strength of evidence among providers. The importance of leadership support to implement guidelines has been documented previously [22], and recent findings also demonstrate the importance of providers’ perceived evidence of infection prevention practices for successful implementation [41]. These are key strengths we found in our evaluation of the early implementation efforts that can be incorporated into future implementation strategies. In addition, we found that the presence of local MPCs, whose role centers on overseeing compliance with the guidelines, was often helpful for implementation; however, a strong group of SCI/D providers could effectively support MRSA prevention activities. This is particularly important in the SCI/D setting given patient needs for interdisciplinary, integrated, and team-based care [38]. Further, future efforts should ensure that guideline dissemination reaches the full range of providers that care for patients with SCI/D and should focus on disseminating guidelines to providers with hands-on contact with patients in inpatient and other SCI/D care settings. We found that nurses were more likely to report full implementation of the guidelines compared to other providers, perhaps due to responsibilities and activities that require nurses to be more aware of the recommended prevention practices. Our findings also showed that guideline awareness was strongest among providers in the initial years of their work experience with SCI/D patients, suggesting a need for enhanced reinforcement of the guidelines targeted to more experienced providers. Next, education around the guidelines should be provided frequently to all SCI/D providers and should focus on SCI/D-specific recommendations for MRSA prevention. In light of these data and the SCI/D community’s need to obtain chronic care from a variety of providers, strategies that focus on education and training for all SCI/D providers are needed. This is supported by a recent study indicating a need for increased education efforts for SCI/D providers to prevent MRSA [27]. Finally, our survey findings revealed perceived barriers to performing key practices included in the SCI/D MRSA prevention guidelines, such as cohorting and isolating MRSA-positive patients, following contact precautions and gowning and gloving in-between patient encounters. Perceived barriers to changing gowns and gloves in-between patients varied by perceived level of implementation, including unique factors to SCI/D, such as unit layout and multi-bed rooms, and should be addressed to enhance ongoing implementation of infection prevention activities.

PARiHS was used to evaluate factors influencing guideline implementation in SCI/D Centers and to identify implementation strategies that can be used for current and planned efforts. The PARiHS framework was beneficial in systematically building knowledge of the context around guideline implementation in SCI/D Centers, given that data in this area remains limited. Our results illustrate the ways in which implementation in the SCI/D setting is shaped by a combination of contextual, evidence, and facilitation elements and sub-elements; this aligns with recent literature demonstrating that implementation is a facilitated process that rests upon interactions between individuals, evidence, and context [25]. This framework may be useful to structure evaluations of other implementation efforts in SCI/D beyond infection prevention. Further theory-guided and mixed method studies are needed that examine the adoption of prevention guidelines for other HAIs in SCI/D to build a knowledge base around the impact of SCI/D-specific characteristics on implementation of infection prevention guidelines.

Several study limitations should be noted. Our survey response rate was lower than expected, where physicians had the lowest response rate. Lower physician response rates have also been noted in prior survey studies examining perceptions of clinical guidelines [22, 42]. Additionally, qualitative interviews were conducted with a relatively small group of providers. As a result, our findings may not be applicable to other providers’ practices and their experiences at VA SCI/D Centers. Finally, the cross-sectional nature of the research design, reliance on self-reported data, and recruitment of provider interviews through the SCI/D Chiefs might have presented study biases. However, the need to identify members with direct experience related to MRSA efforts in the SCI/D Centers was necessary and a means to developing a purposive sample [27, 43].

## Conclusions

Recent findings demonstrate that the VA MRSA Prevention Initiative has been successful and that MRSA infection rates in VA SCI/D Centers have declined [7]. A key step in developing and maintaining effective prevention strategies is understanding factors that influenced early implementation. Facilitators for implementing the SCI/D MRSA prevention guidelines in the VA included enhanced leadership support and provider education around the guidelines, improved dissemination of the guidelines that reached all SCI/D providers, and strong perceived evidence supporting the guidelines. To successfully implement infection prevention guidelines in SCI/D settings, providers and researchers should ensure that (1) guideline dissemination reaches the full range of SCI/D providers, including those working in inpatient, outpatient, and other care settings (2) provider education is frequent and systematic, (3) leadership support is strong, and (4) barriers unique to the recommendations are addressed. Findings from this study contribute to the evidence base around implementation of SCI/D MRSA prevention guidelines in VA SCI/D Centers and can be used to enhance infection prevention and implementation efforts in SCI/D beyond MRSA as well as in other specialty care populations.

## Additional files

Additional file 1:
**VA MRSA SCI Guidelines.** Guidelines for Implementation of MRSA Prevention Initiative in the Spinal Cord Injury Centers. Guidelines for the implementation of the MRSA Prevention Initiative in Spinal Cord Injury Centers, released July 24, 2008. Additional file 2: Appendix A.
**Table of study survey and interview questions mapped to PARiHS elements and sub-elements. Survey and interview questions mapped to the PARiHS framework elements and sub-elements.**
Additional file 3: Appendix B.
**Table of key qualitative themes mapped to PARiHS elements and sub-elements. Key qualitative themes mapped to the PARiHS framework elements and sub-elements.**

## Abbreviations

ASC: active surveillance and screening culture
HAI: hospital-acquired infection
MPC: methicillin-resistant *Staphylococcus aureus* prevention coordinator
MRSA: methicillin-resistant *Staphylococcus aureus*
PARiHS: Promoting Action on Research Implementation in Health Systems
SCI/D: Spinal Cord Injury and Disorder
VA: Veterans Affairs
