# Supplementary material for: Evaluating implementation of methicillin-resistant Staphylococcus aureus (MRSA) prevention guidelines in spinal cord injury centers using the PARIHS framework: a mixed methods study
Source: Implement Sci. 2015 Sep 9;10:130. doi: 10.1186/s13012-015-0318-x (PMC4564999; doi:10.1186/s13012-015-0318-x)
Supplement: Additional file 1: — VA MRSA SCI Guidelines. Guidelines for Implementation of MRSA Prevention Initiative in the Spinal Cord Injury Centers. Guidelines for the implementation of the MRSA Prevention Initiative in Spinal Cord Injury Centers, released July 24, 2008. [file 13012_2015_318_MOESM1_ESM.pdf]

Guidelines for Implementation of MRSA Prevention Initiative in the Spinal Cord Injury Centers

Released July 24, 2008

1. **PURPOSE:** To establish guidelines for the management of patients in Spinal Cord Injury Centers (SCICs) who are colonized or infected with methicillin-resistant *Staphylococcus aureus* (MRSA) which are consistent with the MRSA Prevention Initiative (VHA Directive 2007-002) and the unique needs of veterans with spinal cord injury and disorders (SCI&D). This document addresses the unique aspects of MRSA management in a specialty care setting with high prevalence, increased risk due to co-morbidities, a mixed inpatient population of short and long stays, while maintaining SCI Center access to care for the total population served.

2. **BACKGROUND**: Implementation of the MRSA Prevention Initiative in the Spinal Cord Injury Centers balances the need to prevent and control the spread of MRSA with the overall mission of the SCICs to support, promote, and maintain the health, independence, quality of life, and productivity of veterans with spinal cord injury and disorders (SCI&D). This mission is accomplished by providing state-of-the-art rehabilitation, sustaining medical and surgical care, psychological, social, and vocational care, and patient and family education in the SCICs.

   These guidelines will follow the four general components of the VHA MRSA Prevention Initiative: active surveillance screening, use of contact precautions that accommodate the unique nature of the SCI Center setting, aggressive use of hand hygiene, and cultural change.

3. **RESPONSIBILITIES**

   A. Spinal Cord Injury Center Chiefs will be responsible for the implementation of the MRSA Initiative in the Spinal Cord Injury Center within the context of the overall patient-centered goals to maximize rehabilitation and recovery, function, overall wellness, and independence.
   B. The SCI interdisciplinary team will review these guidelines, support their application, and work under the direction of the SCI Chief in collaboration with the MRSA Prevention Coordinator and Infection Control staff for their implementation.
   C. Staff will actively promote information and will educate patients, family members, visitors and other staff about MRSA and the VHA MRSA Prevention Initiative, in particular the use of Hand Hygiene as one of the best ways to prevent transmission of infectious organisms.
   D. Staff will actively teach and strongly promote the importance and adherence to Contact Precautions when patients are colonized or infected with MRSA.
   E. Staff will promote and implement this guideline whenever possible, balancing the needs of the veterans with SCI and overall SCIC function with the prevention and control of MRSA.

4. **PROCEDURES**
   A. **General considerations.**

1. These procedures are general guidelines and parameters. These guidelines apply to day-to-day operations and are not intended for clusters, outbreaks or increases in MRSA rates which will likely require more intensive intervention.
2. An active educational program and educative materials will be used for staff, patients, family members, surrogates, and caregivers. This education will include but is not limited to MRSA transmission, the risk of infection, and the importance of screening, contact precautions, hand hygiene, and adherence with MRSA prevention practices.
3. As part of good medical practice, the practitioner must discuss these measures with the patient prior to screening, including the nature, purpose, and consequences of the proposed screening. The practitioner must indicate in a signed, dated and timed progress note that this communication occurred.
4. Staff will promote and implement this guideline whenever possible, but not to the exclusion of any patient admission to the SCI Center and overall unit functioning.

B. **Active surveillance screening.**

1. At the beginning of the MRSA Initiative, active surveillance screening of the nares will be conducted on all current inpatients not already known to be colonized or infected with MRSA in the previous 12 months.
2. All patients who have pressure ulcers and are not known to be colonized or infected with MRSA in the wounds will have the wounds swabbed and screened for MRSA.
3. Active surveillance screening of the nares will be done on all previously MRSA-negative patients on admission, transfer, and discharge.
4. Any patient testing positive for MRSA from any specimen on active surveillance screening and/or from clinical culture shall be considered positive for MRSA unless they subsequently meet criteria set forth in the Recommendations for Discontinuance of Contact Precautions, released by the Expert Panel of the VHA MRSA Taskforce.
5. MRSA-negative hospital inpatients will be re-screened at least every 6 months during continuous admission.

C. **Contact Precautions.**

1. Patients found to be colonized or infected with MRSA will be maintained in Contact Precautions.
2. A Contact Precautions sign will be placed on the door of the patient's room or follow facility communication plan to identify MRSA positive patients. When patients leave their room for scheduled appointments, treatments, social activities or events as described in the plan of care, the receiving service should be aware of the MRSA positive status to assure proper continuity of contact precautions. The communication plan should focus on clear communication while maintaining the dignity of each patient.
3. Private rooms and cohorting with other MRSA positive patients in Contact Precautions are encouraged if beds are available. Questions about patient placement should be directed to the local Infection Control Professional or MRSA Prevention Coordinator.
4. Staff
    a. Gloves
        i. Staff will perform hand hygiene before donning gloves.

        ii. Gloves will be worn whenever touching the patient's intact skin, secretions, wounds, blood products, catheters, or surfaces and articles in close proximity to the patient (e.g., medical equipment, bed rails). Don gloves upon entry into the patient's room if direct contact is anticipated.
        iii. Staff will wash hands after removing gloves prior to leaving the room.
    b. Gowns
        i. Staff will wear a gown whenever anticipating that clothing will have direct contact with the patient, potentially-contaminated environmental surfaces in the immediate area of the patient, or equipment in direct contact with the patient.
        ii. Don gown upon entry prior to contact with the patient (e.g., upon entry into the room).
        iii. Remove gown before leaving the patient care environment.
        iv. Staff will wash hands after removing the gloves and gown.

5. Patient
    a. The patient will be encouraged to perform hand hygiene frequently throughout the day including before leaving the room, before and after urinary catheterization and bowel programs, before eating, before and after using equipment, participating in group activities, and visiting other patients. Staff should assess and assist patients who may need assistance with hand hygiene due to impairments (e.g., paralysis) that result from a spinal cord injury.
    b. Patients should bathe or be bathed every day, if possible.
    c. Patients should dress in clean clothes each day, if possible.
    d. Wounds and secretions must be contained prior to leaving the room.
6. Visitors
    a. Visitors are not required to wear gowns and gloves EXCEPT in individual circumstances when providing direct care, when moving from room to room visiting multiple patients, or where other special circumstances dictate this as prudent.
    b. Visitors will be instructed about hand hygiene before and after contact with each patient. If gloves or gowns are used by the visitors, hand hygiene must be performed upon removal of gloves and gowns and prior to leaving the patient's environment.
7. Environmental measures and equipment
    a. Disposable non-critical patient care items or patient-dedicated use of such equipment should be implemented whenever possible.
    b. If common use of equipment for multiple patients is unavoidable, clean and disinfect such equipment before use on another patient using standard hospital-approved germicidal disinfectant or wipes.
    c. Standard hospital-approved germicidal disinfectant or wipes will be used in all rooms for frequent cleaning and disinfection of frequently touched surfaces (e.g., bed rails, overbed table, bedside commode, lavatory surfaces, and doorknobs) and equipment in the immediate vicinity of the patient.
8. Escorting, transporting, and services outside of the room

a. Patients may leave their room for any treatment, procedures, recreational, and social activities as long as wounds, secretions, body fluid, and/or drainage is completely and consistently covered and/or contained.
b. Staff are not required to wear gowns or gloves when escorting or supervising patients unless soiling or contamination is anticipated. Therapy services will observe Contact Precautions as detailed above in 4.C.4.

9. Contact Precautions may be discontinued on previously MRSA-positive patients when they have met the criteria described in the VHA MRSA Taskforce Expert Panel Recommendations for Discontinuance of Contact Precautions.

D. **Hand Hygiene**: Proper hand hygiene is the single most effective measure to decrease the transmission of multidrug-resistant organisms including MRSA within the hospital environment.

1. Staff members will be given specific education and training about the importance of proper hand hygiene.
2. Staff working with patients will wash their hands or use an alcohol-based hand rub before, and after having direct contact with patients.
3. Staff will actively teach and strongly promote hand hygiene among all patients, staff and visitors regardless of MRSA status. This guideline recognizes that some patients with a spinal cord injury may need assistance with hand hygiene due to impairments (e.g., paralysis) that result from a spinal cord injury. As part of routine care, patients' ability to perform hand hygiene should be assessed on an ongoing basis, and assistance provided by staff as necessary.
   a. All patients will be instructed to perform hand hygiene when leaving their room, prior to meals, after catheterizing and toileting, and after touching any body fluids and secretions.
   b. Visibly soiled hands must be washed with soap and water.

E. **Application of Cultural Change.**

It is the intent of this initiative to interrupt the chain of transmission of MRSA and thereby decrease the number of patients at risk for MRSA infection or colonization. Spinal Cord Injury Centers are responsible to define and implement appropriate precautions to prevent the spread of MRSA yet maintain flexibility in order to meet the needs of veterans with SCI&D. SCI staff will be educated about general aspects of MRSA, MRSA prevention in VA, and the specific issues in the SCI population. In keeping with the tenets of the MRSA Prevention Initiative cultural change, SCI staff will be actively engaged in, and work with the facility leadership, MRSA prevention coordinator, and other staff to implement changes that prevent the transmission of MRSA.

REFERENCES:

Association of Professionals in Infection Control and Epidemiology (APIC) Guide to the Elimination of Methicillin-Resistant Staphylococcus aureus (MRSA) Transmission in Hospital Settings 2007. http://www.apic.org/scriptcontent/custom/secure/mrsa_elim_guide.cfm.

Centers for Disease Control and Prevention. Management of Multidrug-Resistant Organisms in Healthcare Settings. http://www.cdc.gov/ncidod/dhqp/pdf/ar/mdroGuideline2006.pdf

Centers for Disease Control and Prevention. Guidelines for Isolation Precautions: Preventing Transmission of Infectious Agents in Healthcare Settings 2007. http://www.cdc.gov/ncidod/dhqp/pdf/isolation2007.pdf

VHA Directive 2007-002, Methicillin-Resistant *Staphylococcus Aureus* Initiative. January 12, 2007. http://vawwl.va.gov/vhapublications/ViewPublicationasp/?pub_ID=1525

VHA CLC Culture Transformation web site: http://www1.va.gov/GeriatricsSHG/page.cfm?pg=75

VHA MRSA Internet web site:http://www.va.gov/pittsburgh/mrsa/index.htm

VHA MRSA Intranet website: http://vaww.va.gov/pittsburgh/mrsa/mrsa_home.htm

VHA Directive 2005-002 Required Hand Hygiene Practices. January 12, 2005. http://vawwl.va.gov/vhapublications/ViewPublication.asp?pub_ID=1214

VHA MRSA Taskforce Expert Panel. Recommendations for Discontinuance of Contact Precautions.

VHA MRSA Taskforce Expert Panel. Recommendations for Decolonization of MRSA.

VHA MRSA Taskforce brochure entitled “Should I Take the MRSA Test? What Veterans needs to know about MRSA testing.”
