# Supplementary material for: Evaluating implementation of methicillin-resistant Staphylococcus aureus (MRSA) prevention guidelines in spinal cord injury centers using the PARIHS framework: a mixed methods study
Source: Implement Sci. 2015 Sep 9;10:130. doi: 10.1186/s13012-015-0318-x (PMC4564999; doi:10.1186/s13012-015-0318-x)
Supplement: Additional file 2: Appendix A. — Table of study survey and interview questions mapped to PARiHS elements and sub-elements. Survey and interview questions mapped to the PARiHS framework elements and sub-elements. [file 13012_2015_318_MOESM2_ESM.docx]

**Additional File 2_Appendix A.doc**

**Appendix A. Study survey and interview questions mapped to PARiHS elements and sub-elements.**

| PARiHS Element | PARiHS Sub-element | Survey Questions | Semi-structured Interview Questions |
| --- | --- | --- | --- |
| Context | Receptive context | • Have you ever seen the VA guidelines for MRSA prevention in SCI Centers entitled “Guidelines for Implementation of MRSA Prevention Initiative in the Spinal Cord Injury Centers” (SCI/D MRSA prevention guidelines)? | • How familiar would you say that you are with the SCI/D MRSA prevention guidelines? |
|  |  | • How frequently do you indicate in a signed, dated, and timed progress note that communication with a patient about MRSA screening has occurred? | • How did you learn about the guidelines? |
|  |  |  | • Are there particular aspects of the guidelines that you feel are more important than others? |
|  |  |  | • To what extent is infection control staff at the hospital level involved in implementing those guidelines in the SCI/D Center? |
|  |  |  | • Is there one barrier that you can point to that is the most challenging and presents the most difficulty for following good MRSA prevention practices? |
|  |  |  |  |
|  |  |  |  |
|  |  |  |  |
|  | Culture | • To what extent do you agree or disagree that your SCI/D Center/interdisciplinary team works closely with the hospital MRSA Prevention Coordinator (MPC) to prevent transmission of MRSA? |  |
|  |  | • Please indicate to what extent do you agree or disagree with the following statements: |  |
|  |  | The implementation of the SCI/D MRSA prevention guidelines has greatly improved your ability to prevent MRSA transmission to MRSA-negative patients in the SCI unit. |  |
|  |  | The implementation of the SCI/D MRSA prevention guidelines has greatly improved your ability to prevent MRSA infection in all SCI/D patients. |  |
|  |  | • Please indicate how often you practice the following in your SCI/D Center: |  |
|  |  | I encourage visitors/family members to perform hand hygiene before and after all patient contact. |  |
|  |  | I encourage SCI/D patients to perform hand hygiene before and after interacting with other patients. |  |
|  |  | I encourage SCI/D patients with impaired hand function to ask for assistance to perform hand hygiene. |  |
|  |  | I bathe SCI/D patients every day. |  |
|  |  | • Please indicate the extent to which you agree or disagree: |  |
|  |  | When other staff in the SCI/D unit do not clean their hands, I feel comfortable reminding them. |  |
|  | Leadership |  | • How did the SCI/D Chief introduce the guidelines in your SCI/D Center? o Held an all staff meeting o Provided a copy of the guidelines to everyone o Gathered a team together to disseminate the guidelines |
|  | Resources | • Please rate the adequacy of each resource the SCI/D Chief provides to implement the SCI/D MRSA prevention guidelines. | • What resources do you think that you need in order to do a good job of preventing the spread of MRSA in your SCI/D Center? |
|  |  | Staff |  |
|  |  | Training |  |
|  |  | Funding |  |
|  |  | • What are the barriers to cohorting/isolating MRSA-positive patients in your SCI/D Center? (check all that apply) | • To what extent do you feel that you receive the resources you need to prevent the spread of MRSA in your SCI/D Center? |
|  |  | Availability of beds |  |
|  |  | Too many MRSA positive patients |  |
|  |  | Layout of unit |  |
|  |  | Patients refuse or get upset |  |
|  |  | Visitors/family members refuse or get upset |  |
|  |  | Too busy |  |
|  |  | Delayed time to get results |  |
|  |  | Disrupts work flow of unit |  |
|  |  | Other |  |
|  |  | • What are the barriers to patient hand hygiene in your SCI/D Center? (check all that apply) |  |
|  |  | Not enough staff available to assist |  |
|  |  | Inadequate availability of wheelchair accessible sink with soap and water |  |
|  |  | Inadequate availability of touchless soap or hand sanitizer |  |
|  |  | Patient unwilling to perform hand hygiene |  |
|  |  | Other |  |
|  |  | • What are the barriers to changing gown and gloves in-between patients in your SCI/D Center? (check all that apply) |  |
|  |  | SCI/D unit layout |  |
|  |  | Competing demands/Too busy |  |
|  |  | Multi-bed rooms |  |
|  |  | Expense of supplies |  |
|  |  | Availability of supplies (i.e. properly-sized gloves, stocking of gowns on the ward) |  |
|  |  | Staff do not follow the SCI/D Center MRSA Program guidelines |  |
|  |  | Other |  |
| Evidence | Research | • What is your perception of the strength of evidence for prevention of MRSA transmission in SCI patients for the following practices: |  |
|  |  | Gloving and gowning before entry into a MRSA positive patient’s room. |  |
|  |  | Taking off gloves and gown before leaving a patient’s room. |  |
|  |  | That anyone entering a MRSA positive patient’s room should wear gown and gloves. |  |
|  |  | • What is your perception of the strength of evidence regarding handwashing in the preventing the spread of resistant organisms (including MRSA) in SCI/D patients by: |  |
|  |  | Health care workers before and after patient contact |  |
|  |  | Visitors/family members before and after patient contact |  |
|  |  | SCI/D patients |  |
|  |  | SCI/D patients with poor hand function |  |
|  |  | • What is your perception of the strength of evidence that supports these practices in SCI units? |  |
|  |  | Active surveillance (nasal screening, nares swabbing) for MRSA at admission |  |
|  |  | Active surveillance (nasal screening, nares swabbing)for MRSA at transfer to another unit |  |
|  |  | Active surveillance (nasal screening, nares swabbing) for MRSA at discharge |  |
|  |  | Active surveillance for MRSA in wounds or pressure ulcers |  |
|  |  | Rescreening patients for MRSA during long admissions |  |
|  | Clinical experience | • Please read the following statements and indicate the extent to which you agree or disagree with each. |  |
|  |  | I believe that healthcare worker hand hygiene is an effective way to reduce MRSA transmission and the development of MRSA infections in SCI/D patients. | • Are there any “best practices” that you personally have for MRSA prevention, that you have found successful? If so, please explain.  o How did you come to these practices? o How do you feel they map to the established SCI/D MRSA prevention guidelines |
|  |  | I believe that patient hand hygiene is an effective way to reduce MRSA transmission and development of MRSA infections in SCI/D patients. |  |
|  |  | • Given the unique issues of SCI/D patients, how much do you agree or disagree with the following statement regarding MRSA: |  |
|  |  | MRSA colonization can be prevented in SCI/D patients. |  |
|  |  | Transmission of MRSA in hospitalized SCI/D patients can be prevented. |  |
|  | Information from local context |  | • Are you aware of any “best practices” that other providers in the SCI/D center have found successful? If so, please explain. |
| Facilitation | Role | • Aside from the hospital MPC, is there a person or a group of people responsible for implementation of SCI/D MRSA prevention guidelines in your unit? | • What, if anything, has leadership done in your SCI/D Denter to facilitate compliance with the guidelines? |
|  |  |  | • What, in your opinion, could your SCI/D Center, VA, or we as VA researchers be doing to try and alleviate these challenges? |
