# Supplementary material for: Evaluating implementation of methicillin-resistant Staphylococcus aureus (MRSA) prevention guidelines in spinal cord injury centers using the PARIHS framework: a mixed methods study
Source: Implement Sci. 2015 Sep 9;10:130. doi: 10.1186/s13012-015-0318-x (PMC4564999; doi:10.1186/s13012-015-0318-x)
Supplement: Additional file 3: Appendix B. — Table of key qualitative themes mapped to PARiHS elements and sub-elements. Key qualitative themes mapped to the PARiHS framework elements and sub-elements. [file 13012_2015_318_MOESM3_ESM.docx]

**Additional File 3_Appendix B.doc**

**Appendix B. Key qualitative themes mapped to PARiHS elements and sub-elements**.

| PARiHS Element | PARiHS Sub-element | Emerging Qualitative Themes | Description/Example |
| --- | --- | --- | --- |
| Context | Receptive context | Guideline dissemination among SCI/D provider groups | Dissemination of guidelines to introduce guidelines to SCI/D providers did not reach all provider groups |
|  |  | SCI/D provider awareness and familiarity with guidelines | Awareness of and familiarity with guidelines varied across SCI/D provider groups |
|  |  | Provider strategies to encourage patient hand hygiene | Provider efforts to promote hand hygiene among bed-bound SCI/D patients who perform self-care by providing hand sanitizers at patient bedside |
|  | Culture | SCI/D Center facility differences | Prevailing beliefs among some providers that SCI/D Centers are more lenient in implementing guidelines compared to acute care settings |
|  | Evaluation | Providing feedback to SCI/D providers | Providers report receiving individual- and system-level feedback regarding their implementation of the guidelines and prevention practices |
| Evidence | Research | Perceived importance and strength of evidence underlying guidelines | Providers perceived strong evidence and believed hand hygiene, contact precautions and active surveillance practices to be especially important for MRSA prevention |
|  | Clinical experience | Perceptions of MRSA colonization, infection and prevention | Based on providers' own clinical experiences, providers perceived contact precautions and active surveillance to be especially relevant for MRSA prevention |
|  | Information from local context | Lack of local evidence to support prevention practices | Some providers described the lack of local evidence available to support prevention practices from guidelines (eg. Lack of data on ‘success rates’ for swabbing patient wounds) |
| Facilitation | Role | Leadership efforts to enable implementation | Leadership efforts to promote/monitor guideline adherence (ie. keeping contact precaution/personal protective equipment stocked outside patient rooms for convenient provider use; anonymously monitoring hand washing) |
|  |  | Involvement of SCI/D and hospital leadership to enable implementation | Use of internal and external agents (eg. Infection Control team, MPCs) to support guideline implementation |
|  |  | Educational practices and training for providers | Use of didactic and traditional training approaches to teaching to educate providers about the guidelines and facilitate implementation |
